# Regulatory T-cell therapy approaches

**DOI:** 10.1093/cei/uxac078

**Published:** 2022-08-12

**Authors:** Oliver McCallion, Merve Bilici, Joanna Hester, Fadi Issa

**Affiliations:** Translational Research Immunology Group, Nuffield Department of Surgical Sciences, University of Oxford, Oxford, UK; Translational Research Immunology Group, Nuffield Department of Surgical Sciences, University of Oxford, Oxford, UK; Translational Research Immunology Group, Nuffield Department of Surgical Sciences, University of Oxford, Oxford, UK; Translational Research Immunology Group, Nuffield Department of Surgical Sciences, University of Oxford, Oxford, UK

**Keywords:** regulatory T cell, adoptive cell therapy, gene editing, CAR-Treg, tolerance

## Abstract

Regulatory T cells (Tregs) have enormous therapeutic potential to treat a variety of immunopathologies characterized by aberrant immune activation. Adoptive transfer of *ex vivo* expanded autologous Tregs continues to progress through mid- to late-phase clinical trials in several disease spaces and has generated promising preliminary safety and efficacy signals to date. However, the practicalities of this strategy outside of the clinical trial setting remain challenging. Here, we review the current landscape of regulatory T-cell therapy, considering emergent approaches and technologies presenting novel ways to engage Tregs, and reflect on the progress necessary to deliver their therapeutic potential to patients.

## Teaser

Regulatory T cells (Tregs) have enormous therapeutic potential to treat a variety of immunopathologies characterized by aberrant immune activation. However, the practicalities of Treg cell therapy outside of the clinical trial setting remain challenging. Here, we review the current landscape of Treg cell therapy, considering emergent approaches and technologies presenting novel ways to engage Tregs, and reflect on the progress necessary to deliver their therapeutic capabilities to patients.

## Introduction

Regulatory T cells (Tregs) play a crucial role in the maintenance of immune homeostasis and antigen tolerance. Abnormalities associated with their number and function have been implicated in the development of a wide range of diseases [1]. Given their unique ability to powerfully control aberrant immune responses, there has been significant interest in targeting Tregs therapeutically for pathologies such as autoimmune diseases and transplant rejection. To date, Treg therapeutic approaches have predominantly comprised either traditional small-molecule or biologic administration directly to patients, or more innovatively through adoptive cellular therapy—so-called “living drugs”—where live Tregs are infused directly into patients. Both approaches have generated exciting glimpses into potential efficacy and continue to progress through clinical evaluation. However, both approaches also have limitations that, as they stand, may limit wider adoption of the therapy. Here, we discuss current Treg cell therapy approaches, consider the barriers and limitations currently faced, and reflect on new therapeutic and technological approaches to enhance their specificity, stability, and applicability.

## Treg biology

### Classification

Tregs comprise a specific subpopulation of CD4^+^ T cells with immunosuppressive function, broadly characterized by their high expression of the IL-2 receptor α-chain (CD25), low expression of the IL-7 receptor (CD127), and expression of the Treg master transcription factor FOXP3. Tregs comprise 5–10% of circulating CD4^+^ lymphocytes and exhibit considerable heterogeneity [2]. They may be classified as either thymus-derived (tTregs or naturally occurring nTregs) or peripherally-derived Tregs (pTregs) [3] according to their site of origin. In the thymus, the strength and duration of the T-cell receptor (TCR) signal commit precursors to a regulatory lineage [4], whilst pTregs differentiate from naïve CD4^+^ T cells following TCR stimulation in the presence of TGF-β and IL-2 in the periphery. Human Tregs may also be classified as naïve (nTregs), effector memory (emTregs), or central memory (cmTregs) based on their differentiation status [5]. Naïve Tregs have not encountered their cognate antigen and reside in secondary lymphoid organs (SLO); on antigen encounter, they become activated and differentiate into cmTregs. Once activated, cmTregs differentiate into emTregs by migrating out of SLOs into the peripheral circulation or tissue [6]. Naïve Tregs have limited immunosuppressive capacity and typically express CD45RA [7], whilst CD45RO is expressed by memory Treg subsets [5]. Each subset also displays unique characteristics in terms of its metabolism and migratory capacity, which may potentially be leveraged therapeutically. For example, naïve Tregs are more dependent on IL-2 for survival compared with effector Tregs which rely on ICOS and IL-7 signalling [8]. The migratory marker L-selectin (CD62L) is expressed by cmTreg and is required for SLO retention. The chemokine receptor CCR7 is also important to distinguish Treg subsets, with naïve Tregs expressing a high level of CCR7 which is downregulated by effector and memory subsets whilst the adhesion receptor CD44 is expressed by memory and effector subsets and correlates with FOXP3 expression and suppressive ability [9]. As phenotype and function are intimately related, it is likely that therapies may in future capitalize on the variability in phenotype and differentiation status to, for example, localize highly suppressive subsets to specific anatomic niches.

### Suppressive mechanisms

Tregs both directly and indirectly suppress CD4^+^ and CD8^+^ T cell, B cell, dendritic cell (DC), macrophage, and natural killer (NK) cell proliferation and function [10]. Contact dependent mechanisms include the expression of granzymes and perforin [11], which directly disrupt target effector cell membranes resulting in apoptosis, and through the expression of inhibitory molecules, for example, CTLA-4 and PD-1 [12, 13]. CTLA-4 and PD-1 interact with CD80/86 and PD-L1 receptors on antigen presenting cells (APCs) respectively, resulting in APC suppression and indirect inhibition of T conventional cells [14]. Contact independent mechanisms include secretion of immunomodulatory cytokines such as IL-10, TGFβ, [13] and IL-35 [15] that suppress conventional T cells and NK cells. In addition, Tregs modulate the extracellular environment to be resistant to effector activation. For example, Tregs express the ectoenzymes CD39 and CD73 which dephosphorylate extracellular ATP to adenosine resulting in effector T-cell suppression [16]. In addition, Tregs express the trimeric IL-2 receptor comprising IL-2Rβ, IL-2Rγ, and the high-affinity IL-2Rα chain (CD25), in contrast to effectors which express a lower-affinity dimeric IL-2 receptor. As Tregs do not produce IL-2 they may act as an IL-2 sink depriving IL-2 from effector populations thereby inhibiting their function [17].

## Treg cell therapy: current approaches

Strategies to enhance Treg number and function provide a more targeted alternative to current therapeutics which rely on generalized non-specific immunosuppression. Numerous preclinical models have demonstrated the significant potential of this approach to treat multiple pathologies including autoimmune-mediated diseases [18–22], graft versus host disease (GvHD) [23], and induce tolerance following solid organ transplantation [24–27]. At present, administration of *ex vivo* expanded Tregs to patients is the most commonly adopted approach [28].

### Adoptive Treg transfer approaches

**Figure 1:**
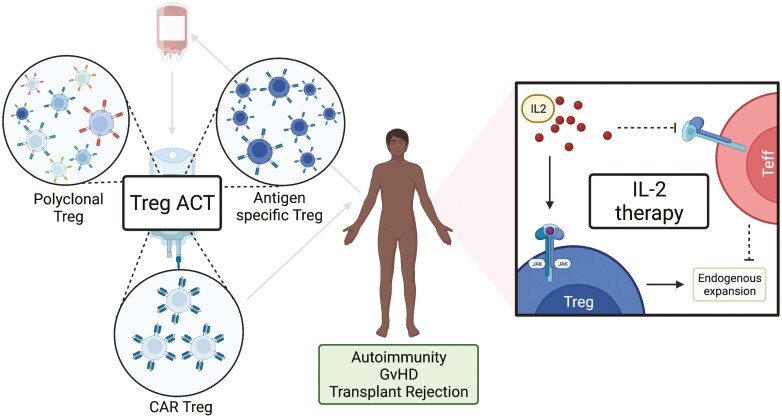
Current approaches to Treg therapy. A schematic overview of the general Treg therapy approaches that have progressed into clinical trials, created with biorender.com. The left-hand panel illustrates adoptive cellular therapy (ACT) approaches starting with donation of autologous whole blood or a leukapheresis product from which the cell therapy is generated. Broadly, Treg ACTs may be classified as: polyclonal, indicating a broad TCR repertoire within the product; antigen specific, indicating a restricted TCR repertoire; or as Tregs transduced with a CAR comprising an ScFv towards a single antigen. The manufactured product is then infused back to the patient donor. The right-hand panel illustrates the approach taken to preferentially expand endogenous Treg *in vivo* through low dose IL-2 therapy. Here, exogenously administered IL-2 is expected to preferentially stimulate and expand Treg expressing the high affinity IL2Rα chain CD25 compared with the lower affinity dimeric IL2R expressed by effector cells.

Adoptive Treg transfer, or more broadly adoptive cellular therapy (ACT), involves a bespoke infusion of Tregs that have been isolated, expanded, and sometimes genetically manipulated, *ex vivo* (Fig. 1). There are several manufacturing protocols, all of which involve collecting starting material from the donor—commonly autologous peripheral blood or an autologous leukapheresis product—from which Tregs are isolated and expanded in culture over multiple rounds of stimulation. Our manufacturing process starts with peripheral blood, from which CD4^+^CD25^+^ lymphocytes are magnetically enriched from PBMCs, and then expanded by TCR stimulation in the presence of IL-2 and rapamycin; the resulting polyclonal product is cryopreserved until infusion [29]. Alternatively, antigen-specific Tregs may be generated by expansion in the presence of professional APCs, most commonly B cells or DCs. However, whilst the adoptive transfer of antigen-specific Tregs may be more potent [30], their precursor frequency is typically low. As such, expanding sufficient cells for therapeutic administration can be challenging particularly as intensive expansion may impair Treg suppressive capacity [31, 32].

#### Treg ACT: clinical experience

Early studies into the safety and feasibility of Treg ACT were predominantly conducted in the setting of haematopoietic stem cell transplantation to prevent or treat GvHD [33, 34], paving the way for trials in autoimmunity and transplantation tolerance to be initiated. In the setting of type 1 diabetes (T1DM), administration of autologous Treg ACT to recently diagnosed children up to 30 × 10^6^/kg was well tolerated and resulted in clinical remission in two-thirds of patients at 12 months [34]. Similarly, treatment of 14 adult patients with recent onset T1DM with autologous Treg ACT up to 26 × 10^8^ per dose in a phase 1 clinical trial was also well tolerated [35, 36]. However, the recently completed phase IIa Sanford Project T-Rex Study (NCT02691247) was reported by the manufacturer Caladrius Biosciences to have missed its primary outcome of improving C-peptide levels at 12 months [37]. Nevertheless, the complete trial outcome is awaited and conceivably may identify subgroups demonstrating an effect given the heterogeneity of the trial population. In the setting of solid organ transplantation, Treg ACT has been administered to both kidney transplant and liver transplant recipients with excellent effect. For example, administration of a Treg enriched product to 10 liver transplant recipients resulted in successful immunosuppression withdrawal in 70% with stable graft function between 5.2 and 6.8 years following cessation [38]. More recently, the ONE study, a global consortium initiating harmonized phase I trials of several cellular therapies, demonstrated the safety of administering polyclonal (up to 10 × 10^6^/kg) Tregs following living donor kidney transplantation, with promising preliminary efficacy signals [39, 40]. Following on from these early data, we have recently initiated the TWO study, a currently recruiting phase 2b clinical trial to examine the ability of autologous Treg ACT to facilitate immunosuppression minimization [41].

### IL-2 therapy approaches

IL-2 was first identified as indispensable for effector T cell survival, proliferation, and function, before its importance in Treg homeostasis was recognized [42]. However, the potential for IL-2 to stimulate both effector and regulatory T cells, in addition to its short half-life (under 15 min) necessitating high-dose administration and frequent, occasionally fatal, dose-limiting toxicities curbed early clinical adoption as a therapeutic to stimulate anti-tumour effector populations [43, 44]. However, preferential expansion of Tregs through exploiting IL-2 signalling differences, namely the high affinity (*K*_d_ ~ 10^−11^ M) of the trimeric IL-2 receptor expressed by Tregs, is under active investigation through administering ultra-low dose IL-2, carefully optimized to promote Treg without effector expansion [45–47]. This approach has been trialled in multiple disease spaces including type 1 diabetes [48], vasculitis [49], GvHD [50], and solid organ transplantation. Ultra-low dosing regimens appear to have a tolerable safety profile, in particular with respect to the incidence of venous thromboembolism which is especially problematic at higher doses [51]. Nevertheless, the therapeutic window of this approach is very narrow, and the potential to inadvertently expand effector populations, and thereby worsen the immunopathology, remains significant. This is well illustrated by recent attempts to use low-dose IL-2 to facilitate immunosuppression withdrawal in liver transplant recipients, where subcutaneous injection of 1 × 10^6^ IU of IL-2 daily resulted in 100% rejection associated with expanded CD25^+^ T effector and NK cell populations [52]. Even when combined with Treg ACT, recombinant low dose IL-2 to treat T1DM results in the expansion of NK, MAIT, and effector CD8^+^ T cell expansion without improvement in metabolic function [53].

## Barriers to routine autologous Treg therapy

Given the safety and early clinical efficacy signals outlined above, consideration of how potential therapeutic benefits may be delivered to larger patient populations outside of the clinical trial environment merits attention. However, scaling up autologous Treg therapy raises several biological, technical, and economic problems requiring innovative solutions. From a biological perspective, the pathology for which Treg ACT is indicated may be associated with intrinsic Treg dysfunction [54–57]; expanding and adoptively transferring dysfunctional Treg in these settings is likely to be of limited benefit. Similarly, not all patients are able to donate a sufficient volume of blood or undergo leukapheresis from which a product may be generated—particularly children and patients with significant co-morbidities. On the technical side, expansion of a sufficient number of Tregs requires several weeks in culture which precludes their acute administration, for example to recipients of deceased donor organs. Equally, the manufactured product may fail to meet the required number, function, purity, and sterility criteria necessary for release and clinical administration. Finally, bespoke cell therapies do not fit well within current small molecule and biologic manufacturing processes. Instead, ACTs require specific facilities and expertise to produce, which are less amenable to economies of scale, a fact that is reflected in significant per-product cost [58]. It is not clear whether these facilities should be centralized or established within academic centres.

## Treg cell therapy: next-generation approaches

**Figure 2:**
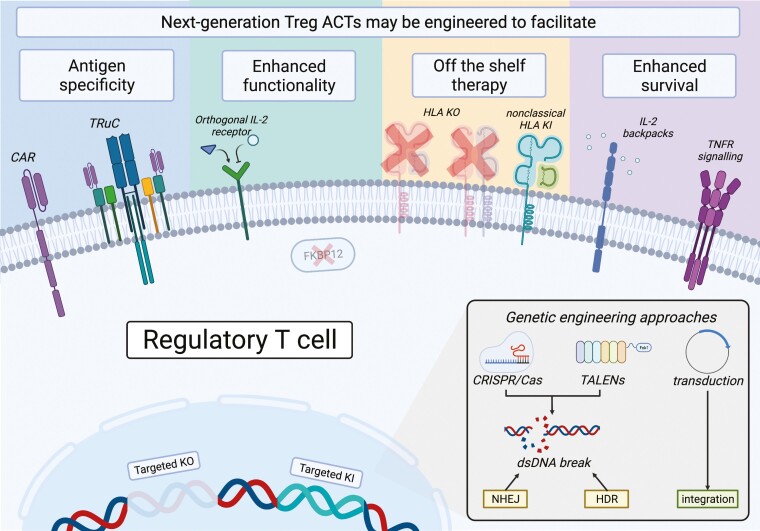
Future approaches to Treg therapy. A schematic overview of selected published experimental approaches adopted to potentiate Treg cell therapy in the pre-clinical setting, created with biorender.com. Broadly, genetic and bioengineering strategies have been investigated to induce antigen specificity in polyclonal Treg products, enhance Treg *in vivo* survival and function through modulating availability and responses to essential Treg homeostatic signals, and to explore use of third party Treg products through inhibiting cell surface expression of HLA, with or without induced expression of additional pro-survival proteins.

Multiple strategies to mitigate current limitations are under active evaluation, leveraging the natural opportunity to apply genome editing and bioengineering technologies to enhance Treg ACT specificity, stability, potency, and source (Fig. 2) [59]. Major advances in this domain have recently been achieved, paving the way for the translation of these technologies into the clinic.

### Improving Treg specificity with Chimeric antigen receptors

Chimeric antigen receptor (CAR) technology has produced several landmark highly-effective novel therapeutics for B-cell precursor acute lymphoblastic leukaemia [60] and diffuse large B cell lymphoma [61]; the same technology demonstrates significant promise to direct Treg cell therapies specifically and potently towards a known antigenic target. Broadly, CARs comprise an extracellular signalling domain, most commonly a single chain variable fragment (scFv), and an intracellular signalling domain, most commonly the CD3ζ activating domain of the T cell receptor that may be potentiated by a co-stimulatory molecule such as CD28 [62]. These are linked by an extracellular hinge region and transmembrane domain. Expression of the wild-type CD28 co-stimulatory domain appears to be particularly important for CAR-Treg generation given that expression of other co-stimulatory domains destabilizes their suppressive function [63, 64]. On the manufacturing side, autologous CAR T cell therapies are produced by either lentiviral or gamma-retroviral transduction of activated autologous T cells with a CAR-encoding vector. Transduced cells are expanded and either used directly or cryopreserved before near-patient thawing and administration [65].

CAR-Tregs present a route to alloantigen-specific tolerance with therapeutic relevance towards numerous pathologies including type 1 diabetes [66], inflammatory bowel disease [67], multiple sclerosis [68], haemophilia [69], vitiligo [70], transplant rejection, and GvHD [71, 72]. In the transplantation space, our predominant strategy has been to exploit frequent donor-recipient human leucocyte antigen (HLA) mismatches by transducing Treg with a CAR specifically targeting donor HLA [73]. In this way, CAR-Treg encounter their cognate antigen only within the allograft resulting in targeted local immunosuppression. This approach has mainly been validated with Tregs expressing CARs targeting HLA-A2, a commonly mismatched antigen between transplant donors and recipients, although only a small number of CARs would be required to cover most potential mismatches. In humanized skin allograft models of transplant rejection, HLA-A2^+^ CAR Tregs infiltrate the graft and prolong its survival in comparison to polyclonal Tregs [74–76]. Equally, HLA-A2^+^ CAR Tregs prevent the development of GvHD following reconstitution with HLA-A2^+^ human PBMCs [77, 78]. However, the ability of CAR-Tregs to induce tolerance appears to be lost when animals are pre-sensitized to donor antigen, suggesting that their ability to control memory responses is limited [76], an important consideration for translational studies. Excitingly, autologous HLA-A2^+^ CAR-Tregs have recently progressed to phase I/IIa clinical trial (STeadfast, NCT04817774). The study team aims to recruit twenty-one unsensitized living donor kidney transplant recipients to receive autologous naïve Treg (CD4^+^CD25^+^CD127^lo/−^CD45RA^+^) transduced with a lentiviral vector encoding an anti-HLA-A*02 CAR in the post-transplant period.

In the pre-clinical setting, efforts to potentiate CAR-Treg stability and function through modulating the construct-encoding sequence also continue. One such approach is to include the sequence encoding IL-10, a key immunosuppressive cytokine, within the HLA-A2^+^ CAR vector, resulting in constitutive or induced IL-10 expression [79]. HLA-A2^+^ CAR-Tregs constitutively expressing IL-10 have an enhanced ability to suppress effector T cell proliferation *in vitro*, although *in vivo* constitutive IL-10 expression is insufficient to maintain a suppressive phenotype [80]. This points towards a significant and more general concern regarding CAR-Tregs, namely *in vivo* stability given chronic antigen exposure—particularly considering the known propensity for Tregs to skew from a regulatory to effector phenotype based on microenvironmental cues [81, 82]. This is illustrated in the case of a high-affinity CAR-Treg with a first-generation scFv recognizing soluble factor 8 (FVIII) in a haemophilia A model which adopted a pro-inflammatory phenotype akin to CAR-Tconv, producing high levels of IL-10, IL-4, and IFN-γ, and ultimately increasing the formation of αFVIII IgG. Interestingly, neither targeted mutation of the CD28 intracellular co-stimulatory domain or constitutive expression of IL-10 in this case is sufficient to restore suppressive phenotype, although reducing the scFv surface expression by transducing a construct where the scFv is complexed to CD3ϵ (a TCR fusion construct, or TRuC) does [80]. Hitting the ‘goldilocks zone’ when it comes to TCR signal strength and duration is of critical importance in realizing the benefit of antigen specific Treg suppression. Incorporating safety features such as suicide switches or molecules that can be targeted for depletion may ultimately be required to mitigate the plasticity risk.

### Genome editing Treg ACTs

The capacity to edit the genome of primary human cells has historically been limited, with the major available technologies—zinc-finger nucleases (ZFNs) and transcription activator-like effector nucleases (TALENs)—being expensive, difficult to synthesize, and having limited delivery vectors [83]. The discovery of the CRISPR (clustered regularly interspaced short palindromic repeats)/Cas9 (CRISPR associated protein 9) system, a component of the bacterial adaptive immune system, has however massively increased the ease and availability by which sequence-specific double stranded DNA (dsDNA) breaks can be introduced [84]. In the CRISPR/Cas9 system, a guide RNA (gRNA) sequence directs the Cas9 protein to its complementary genomic sequence. HNH and RuvC-like nuclease domains then introduce precise dsDNA breaks in the complementary and non-complementary strands respectively which are repaired by one of two endogenous repair mechanisms: non-homologous end joining (NHEJ), an error-prone but rapid process that often introduces insertions or deletions (INDEL) into the target sequence resulting in inactivating knock-out (KO) mutations; or homology-directed repair, a precise but slower process, where a DNA template is utilized to *de novo* synthesize the complementary sequence which may be used to knock-in (KI) genes of interest [85]. A key strength of the CRISPR/Cas9 system is its versatility and ease of delivery as gRNA can target any sequence with an upstream Cas9-species-specific protospacer adjacent motif (PAM) resulting in a highly customizable system. Since the first descriptions of utilizing CRISPR/Cas9 to generate precise KOs and KIs in primary human T cells [86, 87], there has been an explosion of interest in editing Tregs to enhance their survival, durability, stability, and function.

#### Gene engineering to potentiate Treg ACT survival

There are several putative targets that may potentiate Treg survival. c-Jun NH_2_-terminal kinase (JNK) signalling is found in multiple cell types and is implicated in diverse and often contrasting cellular functions including proliferation, cytokine production, and apoptosis via, amongst other mechanisms, activation of the AP-1 transcription factor [88–90]. In T cells, JNK signalling has been implicated in co-stimulatory signal integration, increased IL-2 signalling, potentiated effector function, and ordered cell death [91–94]. Interestingly, Tregs with selective siRNA-mediated knockdown of JNK-1 demonstrate superior survival, secondary to enhanced expression of antiapoptotic proteins, in addition to superior suppressive capacity resulting from enhanced production of immunosuppressive cytokines IL-10 and TGF-β and surface expression of LAG-3 [95]. This manifests as an enhanced ability to prevent rejection of allogenic islet cell transplants in mice [95]. Similarly, overexpression of c-Jun (a downstream JNK target) in CAR-T cells renders them resistant to exhaustion, likely mediated through disruption of AP-1-driven expression of exhaustion-associated gene networks [96]. Instead of targeting pathways driving initiation of exhaustion programmes, the same technique has instead been directed towards retaining the stemness of transduced cells. For example, deletion of the DNA methyltransferase-3-alpha (DNMT3A) from T cells prior to retroviral CAR delivery enhances their antigen-specific proliferative capacity and function [97]. This is associated with retained expression of transcription factors that normally become repressed on repeated antigen exposure and retention of a stem-like transcriptional state. However, mutations in DNMT3A are also associated with numerous haematological malignancies [98], which is of particular concern when considering CAR Tregs given that the point is to preserve their cognate antigen, unlike in the case of malignancy where antigenic clearance is desired. The potential for similar edits to result in leukaemia conversion in this setting may therefore be more significant.

Targeted gene edits may be introduced to provide a competitive survival advantage to Treg ACT in specific disease settings. For example, following transplantation, patients require lifelong non-specific pharmacological immunosuppression (IS) to prevent graft rejection. However, ACT administered to immunosuppressed individuals is also therefore susceptible to IS-mediated dysfunction. A mainstay of IS following transplantation is the calcineurin inhibitor tacrolimus, which exerts its immunosuppressive effect through the cytoplasmic protein FKBP12, preventing calcineurin-mediated NFAT (nuclear factor of activated T cells) translocation and transcriptional activation. FKBP12 KO renders T cells resistant to tacrolimus-induced immunosuppression. GMP-compliant CRISPR/Cas9 mediated KO of *fkbp12* in CMV-specific effector T cells results in superior virus-specific effector function and stimulation in the presence of tacrolimus [99]. A similar technique applied to Treg ACTs has significant potential to reduce the effective ACT dose for transplant recipients.

Innovatively, genome editing approaches may also be combined with small molecule therapeutics to modulate signalling responses and promote specific survival of adoptively transferred cells. One strategy to increase the specificity of IL-2 treatment is to *ex vivo* retrovirally transduce Tregs with an orthogonal IL-2 receptor, which retains native IL-2 signalling through STAT5 but responds selectively to an engineered orthogonal IL-2. Importantly, orthogonal IL-2 binds only to the transduced orthogonal receptor and therefore does not stimulate endogenous populations with either the dimeric (βγ) or trimeric (αβγ) IL2R. Following transduction, Tregs remain functional as reflected by their ability to promote donor haematopoietic stem cell engraftment and prolong heart allograft survival in a mixed chimerism model [100]. Orthogonal IL-2/IL-2R systems have successfully been applied to human CAR-T cells [101]. Whilst their relative scarcity may hinder similar attempts to transduce primary human Treg, the potential to modulate Treg number *in vivo* in real-time through altering IL-2 dosing is intriguing, offering the prospect of ‘fine tuning’ ACT function in response to clinical changes.

#### Gene engineering to potentiate Treg ACT number and stability

A second potential application of genome editing is to create lineage-stabilizing KI or KOs to strengthen the durability of the Treg phenotype on encountering a destabilizing inflammatory milieux. The importance of FOXP3, the Treg master transcription factor, is well illustrated by the clinical syndrome IPEX (immunopathology, polyendocrinopathy, enteropathy, X-linked) resulting from a loss-of-function FOXP3 mutation and causing often-fatal autoimmunity from an absent Treg compartment [55, 102–104]. The prospect of inducing expression of FOXP3 and therefore a regulatory phenotype in bulk T cells is an appealing route to overcome the challenges of low precursor frequency. Successful HDR-mediated integration of a promoter into the FOXP3 locus of human CD4^+^ cells has indeed been achieved, which results in the acquisition of an immunosuppressive phenotype. However, these cells are less potently suppressive, requiring a higher edited Treg:effector ratio, and demonstrate key transcriptomic differences from tTreg, notably in the expression of IKZF2 [105]. This is congruent with previous observations that FOXP3 expression is necessary but not sufficient for Treg function [106–108]. Alternative approaches to induce FOXP3 expression include HDR-mediated KI of FOXP3 or forced demethylation of the Treg-specific demethylation region (TSDR) within the FOXP3 promotor. The former has been investigated as a therapeutic strategy for IPEX, by delivering a functional FOXP3 template alongside CRISPR/Cas9 targeting the mutated version, although this approach is likely of limited applicability for developing Treg cellular therapies [109]. The latter strategy of forcing TSDR demethylation, which conceivably is of greater applicability to the Treg ACT field, has unfortunately had limited success to date with successful TSDR demethylation—achieved by localizing the demethylating enzyme TET1 specifically to TSDR by fusion to cas9—not translating into a regulatory phenotype [110, 111]. It is likely that FOXP3 may need to form part of a broader combination of edits to produce functional Tregs, with HELIOS being a potential second candidate. HELIOS is a member of the Ikaros transcription factor family, which exhibit diverse functions within lymphoid cells regulating both lineage development and mature effector functions [112]. Whilst HELIOS function remains incompletely defined within Tregs, its expression has nevertheless been implicated in Treg differentiation [113], stability [114], and suppressive capacity [115]. As such, inducing durable FOXP3 and HELIOS co-expression may provide a more stable edited Treg product. CD4^+^ and CD8^+^ lymphocytes retrovirally transduced with full-length HELIOS and FOXP3 do indeed acquire a regulatory phenotype, although unfortunately they also exhibit destabilized proliferative potential [116].

Others have approached maintaining Treg phenotypic stability by targeting signalling pathways responsible for promulgating phenotypic skewing. For example, PKC-θ acts as a negative feedback loop to restrain Treg suppressive function [117], although its important role in immune-synapse formation, differential physiological effects between T cell subsets, and structurally homologous related proteins render it a challenging target at a systemic level [118]. Specific inhibition of PKC-θ via engineered antibodies tagged with a cell-penetrating peptide mimic, however, results in increased Treg suppressive capacity *in vitro* and potentiates the therapeutic efficacy of adoptively-transferred Treg preventing GvHD development in a humanized xenograft model [119]. Co-opting a similar approach with an editing strategy may sidestep the necessity to systemically administer these therapies and potentiate Treg ACT.

Alternatively, targeting microenvironmental cues at the initiation of their respective signalling cascades may represent a precise method to maintain Treg ACT stability. This technology heralds from the immuno-oncology field, where it has been applied to potentiate effector function within harsh tumour microenvironments. For instance, NK cells can successfully overcome immunosuppressive TGF-β signalling by fusing the extracellular TGF-β receptor with the stimulatory intracellular domain NKG2D, the NK-stimulatory DNAX-activation protein 12 (DAP12), or a synthetic Notch-like receptor [120, 121]. Whilst examples of a similar approach in Tregs are scarce, the principle of converting a pathogenic Treg-destabilizing cue into a lineage-stabilizing intracellular cascade is appealing. However, given that Notch1 signalling appears to skew Treg towards a Th1 phenotype, a distinct approach may be required for Treg ACT [122].

#### Towards allogeneic Treg ACT

Multiple limitations of autologous Treg cell therapy could be overcome by designing and banking an allogeneic “third-party” Treg product available for off-the-shelf administration. However, unmodified alloTreg products, much like unmatched solid organ transplants, are likely to be recognized by the replete recipient immune system. Here, the best-case scenario would be an inefficacious product whilst in the worst-case allogeneic therapy could conceivably result in exacerbation of underlying immunopathology, acute rejection, and sensitization. Genome editing technologies may potentially be leveraged to abrogate allogeneic cell therapy immunogenicity. In the induced pluripotent stem cell (iPSC) field, there has been significant progress towards this aim. Initial approaches created hypoimmunogenic lines by inhibiting MHC class I and II expression, most commonly through disruption of *B2M* encoding the B_2_-microglobulin required for mature MHC I expression, or class II transactivator (*CIITA*) encoding a key component of the transcription factor complex required for MHC II transcription. Unfortunately, in concordance with the ‘missing self’ hypothesis [123], such approaches render iPSCs susceptible to NK-mediated depletion [124]. Strategies to mitigate this include inducing expression of inhibitory proteins such as non-classical HLA [125–127] or CD47 [128] (a transmembrane glycoprotein that inhibits macrophage-mediated phagocytosis), or through selective retention of HLA-C [129]. However, as most protocols to generate Tregs from iPSCs presently involve retroviral transduction of FOXP3 [130, 131], the ability to generate a Treg therapy from hypoimmune iPSCs remains to be established.

### Non-genomic Treg ACT bioengineering

In addition to the genetic-engineering approaches described above, the generation of Treg cell therapies offers an opportunity to apply other bioengineering approaches to potentiate survival and function. A recent innovative approach to equip Tregs with a ‘nanogel backpack’ has been achieved by impregnating a nanogel with engineered IL-2/Fc conjugated to an anti-CD45 antibody and then incubating this construct with Tregs *ex vivo*. Elegantly, nanogel backpacks only release their payload on Treg activation by exploiting the up-regulation of reducing agents expressed on the cell surface following TCR ligation, and the high-affinity Treg IL2Rα rapidly consumes locally released IL-2 minimizing off-population stimulation. Tregs equipped with nanogel backpacks prolong skin graft survival in a humanized mouse model of transplantation more effectively than standard Tregs associated with antigen-specific Treg expansion and an enhanced Treg:Teff ratio within the graft [132]. This approach is particularly appealing given the ease with which it could be integrated into existing workflows for generating Treg ACT.

### Targeting Treg *in vivo*

An alternative attractive approach to harnessing Treg therapeutic potential is to directly and selectively target endogenous Tregs, thereby circumnavigating the technical and costly process of generating a Treg cell therapy product. Such approaches include developing cytokine therapeutics to selectively expand native Treg populations, or by redirecting Tregs towards specific microanatomical niches to specifically localize their immunosuppressive effects.

#### *In vivo* approaches with cytokine-targeting therapeutics

Several approaches to increase the specificity of IL-2 therapy for Treg populations are under preclinical evaluation (comprehensively reviewed in [43]). IL-2 muteins are generated by targeted mutation of specific amino acid residues which disrupt binding of the dimeric IL-2 receptor whilst preserving binding of the trimeric IL-2 receptor. Muteins may be fused to either whole antibodies [133] or antibody fragments [134] to stabilize their pharmacodynamic properties. Alternatively, human recombinant IL-2 may be complexed with a monoclonal antibody or PEGylated to improve its *in vivo* stability [135]. Interestingly, these approaches may be used to bias preferential trimeric IL2R binding, and thereby preferential Treg specificity. Proposed mechanisms for this phenomenon include steric hindrance, where IL-2 is physically blocked from binding to the dimeric receptor, and triggered exchange, where IL-2 is preferentially liberated from the immune complex in the presence of CD25. Conversely, other IL-2 binding antibody clones may conformationally stabilize IL-2 to promote the binding of IL2Rβ [136, 137]. Interestingly, a manufactured IL-2 and CD25 fusion protein unexpectedly expands Tregs selectively through periodically dissociating *trans* dimerization of the trimeric IL2R [138]. More recently agonism of the dimeric IL-2 receptor has been achieved directly with a bispecific antibody and conceivably a similar approach could be taken with a trispecific antibody [139]. However, IL-2 targeting approaches need careful *in vivo* evaluation considering the potential for effector T cells to also express CD25 on encountering their cognate antigen, meaning that even trimeric IL2R specific therapeutics cannot currently exclusively stimulate the Treg compartment.

The tumour necrosis factor receptor superfamily member 25 (TNFRSF25, DR3) has also demonstrated promise as a pre-clinical target to stimulate *in vivo* Treg expansion. Administration of an anti-TNFRSF25 4C12 antibody in mice results in pronounced Treg expansion up to 30–35% of total CD4^+^ T cells in an IL-2 and MHC-class 2 dependent manner [140]. Administration of anti-TNFRSF25 in mouse models of allogeneic heart, skin, and islet cell transplantation also causes massive *in vivo* Treg expansion and prolongs graft survival [141–143]. Similarly, preferential Treg enrichment in HSCT products, achieved by administering anti-TNFRSF25 to donor animals, facilitates recipient engraftment and reduces early post-transplant sequelae [144–146]. Antibody stimulation of a related TNF receptor superfamily member, TNFR2, also results in dose-dependent proliferation of Treg from human peripheral blood [147], whilst a combination of a TNFR2-specific mutein with IL-2 is also able to expand Tregs from mice [148]. However, agonist antibodies remain complex to develop, illustrated by the limited successes targeting other TNRRSF members, and the distribution of both TNFRSF25 and TNFR2 are not restricted to Tregs, presenting potential challenges in therapeutically translating this approach [149].

#### In vivo approaches with biologics and nanoparticles

Specific Treg targeting with more conventional small molecule and protein therapeutics would naturally present fewer translational challenges. An interesting approach to inducing antigen-specific expansion of Tregs has been demonstrated in animal models using antigen-conjugated tolerogenic poly(lactide-co-glycolide) nanoparticles, which significantly prolong islet graft survival and impair effector T cell responses [150]. *In vivo* generation of antigen-specific Tregs is an enticing prospect, although the approach is also contingent on the accurate identification of the pathogenic antigen repertoire; in settings where the antigenic repertoire is likely to comprise multiple peptides that are highly variable between patients, such as the following transplantation, this approach may have limited applicability. Finally, novel Treg differentiators have more recently been investigated, for example, the small polypeptide miPEP31, which transcriptionally represses microRNA-31 a negative regulator of Treg differentiation [151, 152]. However, whilst directly targeting Tregs with small molecules remains an appealing prospect, the translational potential of new approaches requires careful evaluation.

## Summary

Remarkable progress towards harnessing Treg therapeutic potential has been made since their initial description, and innovative new technologies continue to equip scientists with the tools to produce the next generation of Treg cell therapies. There are several directions of travel, and which is most successful remains to be established. However, it is an exciting prospect to envision multiple broadly applicable Treg cell therapy approaches that will facilitate the delivery of their therapeutic potential to patients.

## Data Availability

No novel data were generated for this review and therefore this statement is not applicable.

## Abbreviations

ACT: adoptive cellular therapy
APCs: antigen presenting cells
CAR: Chimeric antigen receptors
*CIITA*: class II transactivator;
DAP12: DNAX-activation protein 12
dsDNA: double-stranded DNA
gRNA: guide RNA
GvHD: graft versus host disease
iPSC: induced pluripotent stem cell
JNK: c-Jun NH_2_-terminal kinase
KI: knock-in
KO: knock-out
NK: natural killer
PAM: protospacer adjacent motif
pTregs: peripherally-derived Tregs
SLO: secondary lymphoid organs
T1DM: type 1 diabetes
TALENs: transcription activator-like effector nucleases
Tregs: regulatory T cells
TSDR: Treg-specific demethylation region
ZFNs: zinc-finger nucleases

## Permission to reproduce

The article does not contain content requiring permission to reproduce.

## References

[1] Sakaguchi S , Mikami, Wing JB, Tanaka A, Ichiyama K, Ohkura N. Regulatory T cells and human disease. Annu Rev Immunol 2020, 38, 541–66. doi:10.1146/annurev-immunol-042718-041717. 32017635

[2] Dieckmann D , Plottner H, Berchtold S, Berger T, Schuler G. Ex vivo isolation and characterization of Cd4+Cd25+ T cells with regulatory properties from human blood. J Exp Med 2001, 193, 1303–10. doi:10.1084/jem.193.11.1303. 11390437 PMC2193384

[3] Abbas AK , Benoist C, Bluestone JA, Campbell DJ, Ghosh S, Hori S, et al. Regulatory T cells: recommendations to simplify the nomenclature. Nat Immunol 2013, 14, 307–8. doi:10.1038/ni.2554. 23507634

[4] Caramalho I , Nunes-Cabaço H, Foxall RB, Sousa AE. Regulatory T-cell development in the human thymus. Front Immunol 2015, 6, 395. doi:10.3389/fimmu.2015.00395. 26284077 PMC4522873

[5] Miyara M , Yoshioka Y, Kitoh A, Shima T, Wing K, Niwa A, et al. Functional delineation and differentiation dynamics of human CD4+ T cells expressing the FoxP3 transcription factor. Immunity 2009, 30, 899–911. doi:10.1016/j.immuni.2009.03.019. 19464196

[6] Gratz IK , Rosenblum MD, Maurano MM, Paw JS, Truong HA, Marshak-Rothstein A, et al. Cutting edge: self-antigen controls the balance between effector and regulatory T cells in peripheral tissues. J Immunol 2014, 192, 1351–5. doi:10.4049/jimmunol.1301777. 24442443 PMC3925257

[7] Seddiki N , Santner-Nanan B, Tangye SG, Alexander SI, Solomon M, Lee S, et al. Persistence of naive CD45RA+ regulatory T cells in adult life. Blood 2006, 107, 2830–8. doi:10.1182/blood-2005-06-2403. 16332974

[8] Gratz IK , Truong HA, Yang SH, Maurano MM, Lee K, Abbas AK, et al. Cutting Edge: memory regulatory t cells require IL-7 and not IL-2 for their maintenance in peripheral tissues. J Immunol 2013, 190, 4483–7. doi:10.4049/jimmunol.1300212. 23543753 PMC3660612

[9] Firan M , Dhillon S, Estess P, Siegelman MH. Suppressor activity and potency among regulatory T cells is discriminated by functionally active CD44. Blood 2006, 107, 619–27. doi:10.1182/blood-2005-06-2277. 16179372 PMC1895617

[10] Shevach EM. Mechanisms of foxp3+ T regulatory cell-mediated suppression. Immunity 2009, 30, 636–45. doi:10.1016/j.immuni.2009.04.010. 19464986

[11] Barthlott T , Moncrieffe H, Veldhoen M, Atkins CJ, Christensen J, O’Garra A, et al. CD25+ CD4+ T cells compete with naive CD4+ T cells for IL-2 and exploit it for the induction of IL-10 production. Int Immunol 2005, 17, 279–88. doi:10.1093/intimm/dxh207. 15684039

[12] Gondek DC , Lu LF, Quezada SA, Sakaguchi S, Noelle RJ. Cutting edge: contact-mediated suppression by CD4+CD25+ regulatory cells involves a granzyme B-dependent, perforin-independent mechanism. J Immunol 2005, 174, 1783–6. doi:10.4049/jimmunol.174.4.1783. 15699103

[13] Nakamura K , Kitani A, Strober W. Cell contact-dependent immunosuppression by CD4(+)CD25(+) regulatory T cells is mediated by cell surface-bound transforming growth factor beta. J Exp Med 2001, 194, 629–44. doi:10.1084/jem.194.5.629. 11535631 PMC2195935

[14] Gravano DM , Vignali DA. The battle against immunopathology: infectious tolerance mediated by regulatory T cells. Cell Mol Life Sci 2012, 69, 1997–2008. doi:10.1007/s00018-011-0907-z. 22205213 PMC3353028

[15] Collison LW , Workman CJ, Kuo TT, Boyd K, Wang Y, Vignali KM, et al. The inhibitory cytokine IL-35 contributes to regulatory T-cell function. Nature 2007, 450, 566–9. doi:10.1038/nature06306. 18033300

[16] Deaglio S , Dwyer KM, Gao W, Friedman D, Usheva A, Erat A, et al. Adenosine generation catalyzed by CD39 and CD73 expressed on regulatory T cells mediates immune suppression. J Exp Med 2007, 204, 1257–65. doi:10.1084/jem.20062512. 17502665 PMC2118603

[17] Chinen T , Kannan AK, Levine AG, Fan X, Klein U, Zheng Y, et al. An essential role for the IL-2 receptor in T(reg) cell function. Nat Immunol 2016, 17, 1322–33. doi:10.1038/ni.3540. 27595233 PMC5071159

[18] Smyk-Pearson SK , Bakke AC, Held PK, Wildin RS. Rescue of the autoimmune scurfy mouse by partial bone marrow transplantation or by injection with T-enriched splenocytes. Clin Exp Immunol 2003, 133, 193–9. doi:10.1046/j.1365-2249.2003.02217.x. 12869024 PMC1808763

[19] Huter EN , Punkosdy GA, Glass DD, Cheng LI, Ward JM, Shevach EM, et al. TGF-beta-induced Foxp3+ regulatory T cells rescue scurfy mice. Eur J Immunol 2008, 38, 1814–21. doi:10.1002/eji.200838346. 18546144 PMC2574868

[20] Kohm AP , Carpentier PA, Anger HA, Miller SD. Cutting edge: CD4+CD25+ regulatory T cells suppress antigen-specific autoreactive immune responses and central nervous system inflammation during active experimental autoimmune encephalomyelitis. J Imm+unol 2002, 169, 4712–6. doi:10.4049/jimmunol.169.9.4712. 12391178

[21] Stephens LA , Malpass KH, Anderton SM. Curing CNS autoimmune disease with myelin-reactive Foxp3+ Treg. Eur J Immunol 2009, 39, 1108–17. doi:10.1002/eji.200839073. 19350586

[22] Mottet C , Uhlig HH, Powrie F. Cutting edge: cure of colitis by CD4+CD25+ regulatory T cells. J Immunol 2003, 170, 3939–43. doi:10.4049/jimmunol.170.8.3939. 12682220

[23] Taylor PA , Lees CJ, Blazar BR. The infusion of ex vivo activated and expanded CD4(+)CD25(+) immune regulatory cells inhibits graft-versus-host disease lethality. Blood 2002, 99, 3493–9. doi:10.1182/blood.v99.10.3493. 11986199

[24] Xia G , He J, Leventhal JR. Ex vivo-expanded natural CD4+CD25+ regulatory T cells synergize with host T-cell depletion to promote long-term survival of allografts. Am J Transplant 2008, 8, 298–306. doi:10.1111/j.1600-6143.2007.02088.x. 18190656

[25] Issa F , Hester J, Goto R, Nadig SN, Goodacre TE, Wood K, et al. Ex vivo-expanded human regulatory T cells prevent the rejection of skin allografts in a humanized mouse model. Transplantation 2010, 90, 1321–7. doi:10.1097/TP.0b013e3181ff8772. 21048528 PMC3672995

[26] Wu DC , Hester J, Nadig SN, Zhang W, Trzonkowski P, Gray D, et al. Ex vivo expanded human regulatory T cells can prolong survival of a human islet allograft in a humanized mouse model. Transplantation 2013, 96, 707–16. doi:10.1097/TP.0b013e31829fa271. 23917725 PMC3864182

[27] Nadig SN , Więckiewicz J, Wu DC, Warnecke G, Zhang W, Luo S, et al. In vivo prevention of transplant arteriosclerosis by ex vivo–expanded human regulatory T cells. Nat Med 2010, 16, 809–13. doi:10.1038/nm.2154. 20473306 PMC2929438

[28] Bottomley MJ , Brook MO, Shankar S, Hester J, Issa F. Towards regulatory cellular therapies in solid organ transplantation. Trends Immunol 2022, 43, 8–21. doi:10.1016/j.it.2021.11.001. 34844848

[29] Fraser H , Safinia N, Grageda N, Thirkell S, Lowe K, Fry LJ, et al. A Rapamycin-based GMP-compatible process for the isolation and expansion of regulatory T cells for clinical trials. Mol Ther Methods Clin Dev 2018, 8, 198–209. doi:10.1016/j.omtm.2018.01.006. 29552576 PMC5850906

[30] Sagoo P , Ali N, Garg G, Nestle FO, Lechler RI, Lombardi G. Human regulatory T cells with alloantigen specificity are more potent inhibitors of alloimmune skin graft damage than polyclonal regulatory T cells. Sci Transl Med 2011, 3, 83ra42–83ra42. doi:10.1126/scitranslmed.3002076. PMC3776382 21593402

[31] Alzhrani A , Bottomley M, Wood K, Hester J, Issa F. Identification, selection, and expansion of non-gene modified alloantigen-reactive Tregs for clinical therapeutic use. Cell Immunol 2020, 357, 104214. doi:10.1016/j.cellimm.2020.104214. 32977154 PMC8482792

[32] Arroyo Hornero R , Georgiadis C, Hua P, Trzupek D, He L-Z, Qasim W, et al. CD70 expression determines the therapeutic efficacy of expanded human regulatory T cells. Commun Biol 2020, 3, 1–17. doi:10.1038/s42003-020-1097-8. 32665635 PMC7360768

[33] Brunstein CG , Miller JS, Cao Q, McKenna DH, Hippen KL, Curtsinger J, et al. Infusion of ex vivo expanded T regulatory cells in adults transplanted with umbilical cord blood: safety profile and detection kinetics. Blood 2011, 117, 1061–70. doi:10.1182/blood-2010-07-293795. 20952687 PMC3035067

[34] Trzonkowski P , Bieniaszewska M, Juścińska J, Dobyszuk A, Krzystyniak A, Marek N, et al. First-in-man clinical results of the treatment of patients with graft versus host disease with human ex vivo expanded CD4+CD25+CD127- T regulatory cells. Clin Immunol 2009, 133, 22–6. doi:10.1016/j.clim.2009.06.001. 19559653

[35] Marek-Trzonkowska N , Myśliwiec M, Dobyszuk A, Grabowska M, Derkowska I, Juścińska J, et al. Therapy of type 1 diabetes with CD4(+)CD25(high)CD127-regulatory T cells prolongs survival of pancreatic islets - results of one year follow-up. Clin Immunol 2014, 153, 23–30. doi:10.1016/j.clim.2014.03.016. 24704576

[36] Bluestone JA , Buckner JH, Fitch M, Gitelman SE, Gupta S, Hellerstein MK, et al. Type 1 diabetes immunotherapy using polyclonal regulatory T cells. Sci Transl Med 2015, 7, 315ra–189. doi:10.1126/scitranslmed.aad4134. PMC4729454 26606968

[37] Biosciences, C. Caladrius Biosciences Reports Top-Line Data for the Phase 2a Sanford Project: T-Rex Trial of CLBS03 for Recent Onset Type 1 Diabetes, 2019. https://ir.caladrius.com/news-releases/news-release-details/caladrius-biosciences-reports-top-line-data-phase-2a-sanford.

[38] Todo S , Yamashita K, Goto R, Zaitsu M, Nagatsu A, Oura T, et al. A pilot study of operational tolerance with a regulatory T-cell-based cell therapy in living donor liver transplantation. Hepatology 2016, 64, 632–43. doi:10.1002/hep.28459. 26773713

[39] Sawitzki B , Harden PN, Reinke P, Moreau A, Hutchinson JA, Game DS, et al. Regulatory cell therapy in kidney transplantation (The ONE Study): a harmonised design and analysis of seven non-randomised, single-arm, phase 1/2A trials. Lancet 2020, 395, 1627–39. doi:10.1016/s0140-6736(20)30167-7. 32446407 PMC7613154

[40] Harden P , Game D, Sawitzki B, Van Der Net J, Hester J, Bushell A, et al. Feasibility, long-term safety and immune monitoring of regulatory T cell therapy in living donor kidney transplant recipients. Am J Transplant 2021, 21 (4), 1603–11. doi:10.1111/ajt.16395. 33171020 PMC7613119

[41] Brook MO , Hester J, Petchey W, Rombach I, Dutton S, Bottomley MJ, et al. Transplantation Without Overimmunosuppression (TWO) study protocol: a phase 2b randomised controlled single-centre trial of regulatory T cell therapy to facilitate immunosuppression reduction in living donor kidney transplant recipients. BMJ Open 2022, 12, e061864. doi:10.1136/bmjopen-2022-061864. PMC9014059 35428650

[42] Malek TR , Yu A, Vincek V, Scibelli P, Kong L. CD4 regulatory T cells prevent lethal autoimmunity in IL-2Rβ-deficient mice. Immunity 2002, 17, 167–78. doi:10.1016/s1074-7613(02)00367-9. 12196288

[43] Hernandez R , Põder J, Laporte KM, Malek TR. Engineering IL-2 for immunotherapy of autoimmunity and cancer. Nat Rev Immunol 2022, 1–15. Epub ahead of print. doi:10.1038/s41577-022-00680-w. 35217787

[44] Fyfe G , Fisher RI, Rosenberg SA, Sznol M, Parkinson DR, Louie AC. Results of treatment of 255 patients with metastatic renal cell carcinoma who received high-dose recombinant interleukin-2 therapy. J Clin Oncol 1995, 13, 688–96. doi:10.1200/JCO.1995.13.3.688. 7884429

[45] Ito S , Bollard CM, Carlsten M, Melenhorst JJ, Biancotto A, Wang E, et al. Ultra-low dose interleukin-2 promotes immune-modulating function of regulatory T cells and natural killer cells in healthy volunteers. Mol Ther 2014, 22, 1388–95. doi:10.1038/mt.2014.50. 24686272 PMC4089007

[46] Seelig E , Howlett J, Porter L, Truman L, Heywood J, Kennet J, et al. The DILfrequency study is an adaptive trial to identify optimal IL-2 dosing in patients with type 1 diabetes. JCI Insight. 2018, 3(19), 1–17. doi:10.1172/jci.insight.99306. PMC6237447 30282826

[47] Todd JA , Evangelou M, Cutler AJ, Pekalski ML, Walker NM, Stevens HE, et al. Regulatory T cell responses in participants with type 1 diabetes after a single dose of interleukin-2: a non-randomised, open label, adaptive dose-finding trial. PLoS Med 2016, 13: e1002139. doi:10.1371/journal.pmed.1002139. 27727279 PMC5058548

[48] Hartemann A , Bensimon G, Payan CA, Jacqueminet S, Bourron O, Nicolas N, et al. Low-dose interleukin 2 in patients with type 1 diabetes: a phase 1/2 randomised, double-blind, placebo-controlled trial. Lancet Diabetes Endocrinol. 2013, 1, 295–305. doi:10.1016/S2213-8587(13)70113-X. 24622415

[49] Saadoun D , Rosenzwajg M, Joly F, Six A, Carrat F, Thibault V, et al. Regulatory T-cell responses to low-dose interleukin-2 in HCV-induced vasculitis. N Engl J Med. 2011, 365, 2067–77. doi:10.1056/nejmoa1105143. 22129253

[50] Koreth J , Matsuoka K-I, Kim HT, Mcdonough SM, Bindra B, Alyea EP, et al. Interleukin-2 and regulatory T cells in graft-versus-host disease. N Engl J Med 2011, 365, 2055–66. doi:10.1056/nejmoa1108188. 22129252 PMC3727432

[51] Mahmoudpour SH , Jankowski M, Valerio L, Becker C, Espinola-Klein C, Konstantinides S, et al. Safety of low-dose subcutaneous recombinant interleukin-2: systematic review and meta-analysis of randomized controlled trials. Sci Rep. 2019, 9, 1–9. doi:10.1038/s41598-019-43530-x. PMC6509335 31073219

[52] Lim T , Ruiz P, Kurt A, Kodela E, Martinez-Llordella M, Tree T, et al. Use of low dose interleukin-2 to expand regulatory T-cells and facilitate the complete discontinuation of immunosuppression in human liver transplantation. Am Transplant Congress. 2019, 19(suppl 3).

[53] Dong S , Hiam-Galvez KJ, Mowery CT, Herold KC, Gitelman SE, Esensten JH, et al. The effect of low-dose IL-2 and Treg adoptive cell therapy in patients with type 1 diabetes. JCI Insight. 2021, 6(18), 1–18. doi:10.1172/jci.insight.147474. PMC8492314 34324441

[54] Gradolatto A , Nazzal D, Truffault F, Bismuth J, Fadel E, Foti M, et al. Both Treg cells and Tconv cells are defective in the Myasthenia gravis thymus: Roles of IL-17 and TNF-α. J Autoimmun 2014, 52, 53–63. doi:10.1016/j.jaut.2013.12.015. 24405842

[55] Bacchetta R. Defective regulatory and effector T cell functions in patients with FOXP3 mutations. J Clin Investig 2006, 116, 1713–22. doi:10.1172/jci25112. 16741580 PMC1472239

[56] Flores-Borja F , Jury EC, Mauri C, Ehrenstein MR. Defects in CTLA-4 are associated with abnormal regulatory T cell function in rheumatoid arthritis. Proc Natl Acad Sci USA 2008, 105, 19396–401. doi:10.1073/pnas.0806855105. 19036923 PMC2614772

[57] Beers DR , Zhao W, Wang J, Zhang X, Wen S, Neal D, et al. ALS patients’ regulatory T lymphocytes are dysfunctional, and correlate with disease progression rate and severity. JCI Insight 2017, 2(5), 1–14. doi:10.1172/jci.insight.89530. PMC5333967 28289705

[58] Abou-El-Enein M , Bauer G, Medcalf N, Volk H-D, Reinke P. Putting a price tag on novel autologous cellular therapies. Cytotherapy 2016, 18, 1056–61. doi:10.1016/j.jcyt.2016.05.005. 27288308

[59] Milward KF , Wood KJ, Hester J. Enhancing human regulatory T cells in vitro for cell therapy applications. Immunol Lett 2017, 190, 139–47. doi:10.1016/j.imlet.2017.08.012. 28823885

[60] Maude SL , Laetsch TW, Buechner J, Rives S, Boyer M, Bittencourt H, et al. Tisagenlecleucel in children and young adults with B-cell lymphoblastic leukemia. N Engl J Med 2018, 378, 439–48. doi:10.1056/nejmoa1709866. 29385370 PMC5996391

[61] Locke FL , Neelapu SS, Bartlett NL, Siddiqi T, Chavez JC, Hosing CM, et al. Phase 1 results of ZUMA-1: a multicenter study of KTE-C19 anti-CD19 CAR T cell therapy in refractory aggressive lymphoma. Mol Ther 2017, 25, 285–95. doi:10.1016/j.ymthe.2016.10.020. 28129122 PMC5363293

[62] Rafiq S , Hackett CS, Brentjens RJ. Engineering strategies to overcome the current roadblocks in CAR T cell therapy. Nat Rev Clin Oncol 2020, 17, 147–67. doi:10.1038/s41571-019-0297-y. 31848460 PMC7223338

[63] Dawson NAJ , Rosado-Sánchez I, Novakovsky GE, Fung VCW, Huang Q, McIver E, et al. Functional effects of chimeric antigen receptor co-receptor signaling domains in human regulatory T cells. Sci Transl Med 2020, 12, eaaz3866. doi:10.1126/scitranslmed.aaz3866. 32817364

[64] Boroughs AC , Larson RC, Choi BD, Bouffard AA, Riley LS, Schiferle E, et al. Chimeric antigen receptor costimulation domains modulate human regulatory T cell function. JCI Insight 2019, 4(8), 1–19. doi:10.1172/jci.insight.126194. PMC6538349 30869654

[65] Vormittag P , Gunn R, Ghorashian S, Veraitch FS. A guide to manufacturing CAR T cell therapies. Curr Opin Biotechnol 2018, 53, 164–81. doi:10.1016/j.copbio.2018.01.025. 29462761

[66] Tenspolde M , Zimmermann K, Weber LC, Hapke M, Lieber M, Dywicki J, et al. Regulatory T cells engineered with a novel insulin-specific chimeric antigen receptor as a candidate immunotherapy for type 1 diabetes. J Autoimmun 2019, 103, 102289. doi:10.1016/j.jaut.2019.05.017. 31176558

[67] Elinav E , Adam N, Waks T, Eshhar Z. Amelioration of colitis by genetically engineered murine regulatory T cells redirected by antigen-specific chimeric receptor. Gastroenterology 2009, 136, 1721–31. doi:10.1053/j.gastro.2009.01.049. 19208357

[68] Fransson M , Piras E, Burman J, Nilsson B, Essand M, Lu B, et al. CAR/FoxP3-engineered T regulatory cells target the CNS and suppress EAE upon intranasal delivery. J Neuroinflammation 2012, 9, 112. doi:10.1186/1742-2094-9-112. 22647574 PMC3403996

[69] Yoon J , Schmidt A, Zhang A-H, Königs C, Kim YC, Scott DW. FVIII-specific human chimeric antigen receptor T-regulatory cells suppress T- and B-cell responses to FVIII. Blood 2017, 129, 238–45. doi:10.1182/blood-2016-07-727834. 28064157 PMC5234219

[70] Mukhatayev Z , Dellacecca ER, Cosgrove C, Shivde R, Jaishankar D, Pontarolo-Maag K, et al. Antigen specificity enhances disease control by tregs in vitiligo. Front Immunol 2020, 11, 1–15. doi:10.3389/fimmu.2020.581433. 33335528 PMC7736409

[71] Pierini A , Iliopoulou BP, Peiris H, Pérez-Cruz M, Baker J, Hsu K, et al. T cells expressing chimeric antigen receptor promote immune tolerance. JCI Insight 2017, 2(20), 1–17. doi:10.1172/jci.insight.92865. PMC5846896 29046484

[72] Macdonald KG , Hoeppli RE, Huang Q, Gillies J, Luciani DS, Orban PC, et al. Alloantigen-specific regulatory T cells generated with a chimeric antigen receptor. J Clin Investig 2016, 126, 1413–24. doi:10.1172/jci82771. 26999600 PMC4811124

[73] Wright S , Hennessy C, Hester J, Issa F. Chimeric antigen receptors and regulatory T cells: the potential for HLA-specific immunosuppression in transplantation. Engineering 2022, 10, 30–43. doi:10.1016/j.eng.2021.10.018.

[74] Boardman DA , Philippeos C, Fruhwirth GO, Ibrahim MAA, Hannen RF, Cooper D, et al. Expression of a chimeric antigen receptor specific for donor HLA class I enhances the potency of human regulatory T cells in preventing human skin transplant rejection. Am J Transplant 2017, 17, 931–43. doi:10.1111/ajt.14185. 28027623

[75] Noyan F , Zimmermann K, Hardtke-Wolenski M, Knoefel A, Schulde E, Geffers R, et al. Prevention of allograft rejection by use of regulatory T cells with an MHC-specific chimeric antigen receptor. Am J Transplant 2017, 17, 917–30. doi:10.1111/ajt.14175. 27997080

[76] Sicard A , Lamarche C, Speck M, Wong M, Rosado-Sánchez I, Blois M, et al. Donor-specific chimeric antigen receptor Tregs limit rejection in naive but not sensitized allograft recipients. Am J Transplant 2020, 20, 1562–73. doi:10.1111/ajt.15787. 31957209

[77] MacDonald KG , Hoeppli RE, Huang Q, Gillies J, Luciani DS, Orban PC, et al. Alloantigen-specific regulatory T cells generated with a chimeric antigen receptor. J Clin Invest 2016, 126, 1413–24. doi:10.1172/JCI82771. 26999600 PMC4811124

[78] Muller YD , Ferreira LMR, Ronin E, Ho P, Nguyen V, Faleo G, et al. Precision engineering of an anti-HLA-A2 chimeric antigen receptor in regulatory T cells for transplant immune tolerance. Front Immunol 2021, 12, 1–15. doi:10.3389/fimmu.2021.686439. PMC8488356 34616392

[79] Mohseni YR , Saleem A, Tung SL, Dudreuilh C, Lang C, Peng Q, et al. Chimeric antigen receptor-modified human regulatory T cells that constitutively express IL-10 maintain their phenotype and are potently suppressive. Eur J Immunol 2021, 51, 2522–30. doi:10.1002/eji.202048934. 34320225 PMC8581768

[80] Rana J , Perry DJ, Kumar SRP, Muñoz-Melero M, Saboungi R, Brusko TM, et al. CAR- and TRuC-redirected regulatory T cells differ in capacity to control adaptive immunity to FVIII. Mol Ther 2021, 29, 2660–76. doi:10.1016/j.ymthe.2021.04.034. 33940160 PMC8417451

[81] Yang XO , Nurieva R, Martinez GJ, Kang HS, Chung Y, Pappu BP, et al. Molecular antagonism and plasticity of regulatory and inflammatory T cell programs. Immunity 2008, 29, 44–56. doi:10.1016/j.immuni.2008.05.007. 18585065 PMC2630532

[82] Komatsu N , Okamoto K, Sawa S, Nakashima T, Oh-Hora M, Kodama T, et al. Pathogenic conversion of Foxp3+ T cells into TH17 cells in autoimmune arthritis. Nat Med 2014, 20, 62–8. doi:10.1038/nm.3432. 24362934

[83] Gaj T , Gersbach CA, Barbas CF. ZFN, TALEN, and CRISPR/Cas-based methods for genome engineering. Trends Biotechnol 2013, 31, 397–405. doi:10.1016/j.tibtech.2013.04.004. 23664777 PMC3694601

[84] Jinek M , Chylinski K, Fonfara I, Hauer M, Doudna JA, Charpentier EA, et al. A Programmable dual-RNA-guided DNA endonuclease in adaptive bacterial immunity. Science 2012, 337, 816–21. doi:10.1126/science.1225829. 22745249 PMC6286148

[85] Yeh CD , Richardson CD, Corn JE. Advances in genome editing through control of DNA repair pathways. Nat Cell Biol 2019, 21, 1468–78. doi:10.1038/s41556-019-0425-z. 31792376

[86] Roth TL , Puig-Saus C, Yu R, Shifrut E, Carnevale J, Li PJ, et al. Reprogramming human T cell function and specificity with non-viral genome targeting. Nature 2018, 559, 405–9. doi:10.1038/s41586-018-0326-5. 29995861 PMC6239417

[87] Mandal PK , Ferreira LM, Collins R, Meissner TB, Boutwell CL, Friesen M, et al. Efficient ablation of genes in human hematopoietic stem and effector cells using CRISPR/Cas9. Cell Stem Cell 2014, 15, 643–52. doi:10.1016/j.stem.2014.10.004. 25517468 PMC4269831

[88] Macián F , López-Rodríguez C, Rao A. Partners in transcription: NFAT and AP-1. Oncogene 2001, 20, 2476–89. doi:10.1038/sj.onc.1204386. 11402342

[89] Cui J , Zhang M, Zhang Y-Q, Xu Z. -H. JNK pathway: diseases and therapeutic potential. Acta Pharmacol Sin 2007, 28, 601–8. doi:10.1111/j.1745-7254.2007.00579.x. 17439715

[90] Leppä S , Bohmann D. Diverse functions of JNK signaling and c-Jun in stress response and apoptosis. Oncogene 1999, 18, 6158–62. doi:10.1038/sj.onc.1203173. 10557107

[91] Su B , Jacinto E, Hibi M, Kallunki T, Karin M, Ben-Neriah Y. JNK is involved in signal integration during costimulation of T lymphocytes. Cell 1994, 77, 727–36. doi:10.1016/0092-8674(94)90056-6. 8205621

[92] Brandt B , Abou-Eladab EF, Tiedge M, Walzel H. Role of the JNK/c-Jun/AP-1 signaling pathway in galectin-1-induced T-cell death. Cell Death Dis 2010, 1, e23–e23. doi:10.1038/cddis.2010.1. 21364631 PMC3032336

[93] Dong C , Yang DD, Tournier C, Whitmarsh AJ, Xu J, Davis RJ, et al. JNK is required for effector T-cell function but not for T-cell activation. Nature 2000, 405, 91–4. doi:10.1038/35011091. 10811224

[94] Conze D , Krahl T, Kennedy N, Weiss L, Lumsden J, Hess P, et al. c-Jun NH2-terminal kinase (JNK)1 and JNK2 have distinct roles in CD8+ T cell activation. J Exp Med 2002, 195, 811–23. doi:10.1084/jem.20011508. 11927626 PMC2193724

[95] Tripathi D , Cheekatla SS, Paidipally P, Radhakrishnan RK, Welch E, Thandi RS, et al. c-Jun N-terminal kinase 1 defective CD4+CD25+FoxP3+ cells prolong islet allograft survival in diabetic mice. Sci Rep 2018, 8, 1–13. doi:10.1038/s41598-018-21477-9. 29459675 PMC5818514

[96] Lynn RC , Weber EW, Sotillo E, Gennert D, Xu P, Good Z, et al. c-Jun overexpression in CAR T cells induces exhaustion resistance. Nature 2019, 576, 293–300. doi:10.1038/s41586-019-1805-z. 31802004 PMC6944329

[97] Prinzing B , Zebley CC, Petersen CT, Fan Y, Anido AA, Yi Z, et al. Deleting DNMT3A in CAR T cells prevents exhaustion and enhances antitumor activity. Sci Transl Med 2021, 13, eabh0272. doi:10.1126/scitranslmed.abh0272. 34788079 PMC8733956

[98] Yang L , Rau R, Goodell MA. DNMT3A in haematological malignancies. Nat Rev Cancer 2015, 15, 152–65. doi:10.1038/nrc3895. 25693834 PMC5814392

[99] Amini L , Wagner DL, Rössler U, Zarrinrad G, Wagner LF, Vollmer T, et al. CRISPR-Cas9-edited tacrolimus-resistant antiviral T cells for advanced adoptive immunotherapy in transplant recipients. Mol Ther 2021, 29, 32–46. doi:10.1016/j.ymthe.2020.09.011. 32956624 PMC7791012

[100] Hirai T , Ramos TL, Lin P-Y, Simonetta F, Su LL, Picton LK, et al. Selective expansion of regulatory T cells using an orthogonal IL-2/IL-2 receptor system facilitates transplantation tolerance. J Clin Investig 2021, 131(8), 1–13. doi:10.1172/jci139991. PMC8059880 33855972

[101] Zhang Q , Hresko ME, Picton LK, Su L, Hollander MJ, Nunez-Cruz S, et al. A human orthogonal IL-2 and IL-2Rβ system enhances CAR T cell expansion and antitumor activity in a murine model of leukemia. Sci Transl Med 2021, 13, eabg6986. doi:10.1126/scitranslmed.abg6986. 34936380 PMC9116279

[102] Asano M , Toda M, Sakaguchi N, Sakaguchi S. Autoimmune disease as a consequence of developmental abnormality of a T cell subpopulation. J Exp Med 1996, 184, 387–96. doi:10.1084/jem.184.2.387. 8760792 PMC2192701

[103] Bennett CL , Christie J, Ramsdell F, Brunkow ME, Ferguson PJ, Whitesell L, et al. The immune dysregulation, polyendocrinopathy, enteropathy, X-linked syndrome (IPEX) is caused by mutations of FOXP3. Nat Genet 2001, 27, 20–1. doi:10.1038/83713. 11137993

[104] Wildin RS. Clinical and molecular features of the immunodysregulation, polyendocrinopathy, enteropathy, X linked (IPEX) syndrome. J Med Genet 2002, 39, 537–45. doi:10.1136/jmg.39.8.537. 12161590 PMC1735203

[105] Honaker Y , Hubbard N, Xiang Y, Fisher L, Hagin D, Sommer K, et al. Gene editing to induce FOXP3 expression in human CD4+ T cells leads to a stable regulatory phenotype and function. Sci Transl Med 2020, 12, eaay6422. doi:10.1126/scitranslmed.aay6422. 32493794

[106] Dominguez-Villar M , Baecher-Allan CM, Hafler DA. Identification of T helper type 1–like, Foxp3+ regulatory T cells in human autoimmune disease. Nat Med 2011, 17, 673–5. doi:10.1038/nm.2389. 21540856 PMC3675886

[107] Miyao T , Floess S, Setoguchi R, Luche H, Fehling HJ, Waldmann H, et al. Plasticity of Foxp3(+) T cells reflects promiscuous Foxp3 expression in conventional T cells but not reprogramming of regulatory T cells. Immunity 2012, 36, 262–75. doi:10.1016/j.immuni.2011.12.012. 22326580

[108] Ohkura N , Sakaguchi S. Transcriptional and epigenetic basis of Treg cell development and function: its genetic anomalies or variations in autoimmune diseases. Cell Res 2020, 30, 465–74. doi:10.1038/s41422-020-0324-7. 32367041 PMC7264322

[109] Goodwin M , Lee E, Lakshmanan U, Shipp S, Froessl L, Barzaghi F, et al. CRISPR-based gene editing enables FOXP3 gene repair in IPEX patient cells. Sci Adv 2020, 6, eaaz0571. doi:10.1126/sciadv.aaz0571. 32494707 PMC7202871

[110] Kressler C , Gasparoni G, Nordström K, Hamo D, Salhab A, Dimitropoulos C, et al. Targeted de-methylation of the FOXP3-TSDR is sufficient to induce physiological FOXP3 expression but not a functional treg phenotype. Front Immunol 2021, 11, 1–13. doi:10.3389/fimmu.2020.609891. PMC7817622 33488615

[111] Wilk C , Effenberg L, Abberger H, Steenpass L, Hansen W, Zeschnigk M, et al. CRISPR/Cas9-mediated demethylation of FOXP3-TSDR toward Treg-characteristic programming of Jurkat T cells. Cell Immunol 2022, 371, 104471. doi:10.1016/j.cellimm.2021.104471. 34954490

[112] Read KA , Jones DM, Freud AG, Oestreich KJ. Established and emergent roles for Ikaros transcription factors in lymphoid cell development and function. Immunol Rev 2021, 300, 82–99. doi:10.1111/imr.12936. 33331000 PMC8015388

[113] Ng MSF , Roth TL, Mendoza VF, Marson A, Burt TD. Helios enhances the preferential differentiation of human fetal CD4+ naïve T cells into regulatory T cells. Sci Immunol 2019, 4, eaav5947. doi:10.1126/sciimmunol.aav5947. 31757834 PMC7340007

[114] Kim H-J , Barnitz RA, Kreslavsky T, Brown FD, Moffett H, Lemieux ME, et al. Stable inhibitory activity of regulatory T cells requires the transcription factor Helios. Science 2015, 350, 334–9. doi:10.1126/science.aad0616. 26472910 PMC4627635

[115] Takatori H , Kawashima H, Matsuki A, Meguro K, Tanaka S, Iwamoto T, et al. Helios enhances treg cell function in cooperation with FoxP3. Arthritis Rheumatol 2015, 67, 1491–502. doi:10.1002/art.39091. 25733061

[116] Seng A , Krausz KL, Pei D, Koestler DC, Fischer RT, Yankee TM, et al. Coexpression of FOXP3 and a Helios isoform enhances the effectiveness of human engineered regulatory T cells. Blood Adv 2020, 4, 1325–39. doi:10.1182/bloodadvances.2019000965. 32259202 PMC7160257

[117] Zanin-Zhorov A , Ding Y, Kumari S, Attur M, Hippen KL, Brown M, et al. Protein kinase C-theta mediates negative feedback on regulatory T cell function. Science 2010, 328, 372–6. doi:10.1126/science.1186068. 20339032 PMC2905626

[118] Tedesco-Silva H , Kho MML, Hartmann A, Vitko S, Russ G, Rostaing L, et al. Sotrastaurin in calcineurin inhibitor-free regimen using everolimus in *De Novo* kidney transplant recipients. Am J Transplant 2013, 13, 1757–68. doi:10.1111/ajt.12255. 23659755

[119] Ozay EI , Shanthalingam S, Sherman HL, Torres JA, Osborne BA, Tew GN, et al. Cell-penetrating anti-protein kinase C theta antibodies act intracellularly to generate stable, highly suppressive regulatory T cells. Mol Ther 2020, 28, 1987–2006. doi:10.1016/j.ymthe.2020.05.020. 32492367 PMC7474270

[120] Wang Z , Guo L, Song Y, Zhang Y, Lin D, Hu B, et al. Augmented anti-tumor activity of NK-92 cells expressing chimeric receptors of TGF-βR II and NKG2D. Cancer Immunol Immunother 2017, 66, 537–48. doi:10.1007/s00262-017-1959-1. 28184969 PMC11028961

[121] Burga RA , Yvon E, Chorvinsky E, Fernandes R, Cruz CRY, Bollard CM. Engineering the TGFβ receptor to enhance the therapeutic potential of natural killer cells as an immunotherapy for neuroblastoma. Clin Cancer Res 2019, 25, 4400–12. doi:10.1158/1078-0432.ccr-18-3183. 31010834 PMC6635028

[122] Charbonnier L-M , Wang S, Georgiev P, Sefik E, Chatila TA. Control of peripheral tolerance by regulatory T cell–intrinsic Notch signaling. Nat Immunol 2015, 16, 1162–73. doi:10.1038/ni.3288. 26437242 PMC4618075

[123] Kärre K. Natural killer cell recognition of missing self. Nat Immunol 2008, 9, 477–80. doi:10.1038/ni0508-477. 18425103

[124] Wang D , Quan Y, Yan Q, Morales JE, Wetsel RA. Targeted disruption of the β2-microglobulin gene minimizes the immunogenicity of human embryonic stem cells. Stem Cells Transl Med 2015, 4, 1234–45. doi:10.5966/sctm.2015-0049. 26285657 PMC4572902

[125] Gornalusse GG , Hirata RK, Funk SE, Riolobos L, Lopes VS, Manske G, et al. HLA-E-expressing pluripotent stem cells escape allogeneic responses and lysis by NK cells. Nat Biotechnol 2017, 35, 765–72. doi:10.1038/nbt.3860. 28504668 PMC5548598

[126] Torikai H , Reik A, Soldner F, Warren EH, Yuen C, Zhou Y, et al. Toward eliminating HLA class I expression to generate universal cells from allogeneic donors. Blood 2013, 122, 1341–9. doi:10.1182/blood-2013-03-478255. 23741009 PMC3750336

[127] Zhao L , Teklemariam T, Hantash BM. Heterelogous expression of mutated HLA-G decreases immunogenicity of human embryonic stem cells and their epidermal derivatives. Stem Cell Res 2014, 13, 342–54. doi:10.1016/j.scr.2014.08.004. 25218797

[128] Deuse T , Hu X, Gravina A, Wang D, Tediashvili G, De C, et al. Hypoimmunogenic derivatives of induced pluripotent stem cells evade immune rejection in fully immunocompetent allogeneic recipients. Nat Biotechnol 2019, 37, 252–8. doi:10.1038/s41587-019-0016-3. 30778232 PMC6419516

[129] Xu H , Wang B, Ono M, Kagita A, Fujii K, Sasakawa N, et al. Targeted disruption of HLA genes via CRISPR-Cas9 generates iPSCs with enhanced immune compatibility. Cell Stem Cell 2019, 24, 566–578.e7. doi:10.1016/j.stem.2019.02.005. 30853558

[130] Haque R , Lei F, Xiong X, Bian Y, Zhao B, Wu Y, et al. Programming of regulatory T cells from pluripotent stem cells and prevention of autoimmunity. J Immunol 2012, 189, 1228–36. doi:10.4049/jimmunol.1200633. 22732595 PMC3401327

[131] Haque M , Lei F, Xiong X, Das JK, Ren X, Fang D, et al. Stem cell–derived tissue-associated regulatory T cells suppress the activity of pathogenic cells in autoimmune diabetes. JCI Insight 2019, 4(7), 1–13. doi:10.1172/jci.insight.126471. PMC6483657 30777937

[132] Eskandari SK , Sulkaj I, Melo MB, Li N, Allos H, Alhaddad JB, et al. Regulatory T cells engineered with TCR signaling–responsive IL-2 nanogels suppress alloimmunity in sites of antigen encounter. Sci Transl Med 2020, 12, eaaw4744. doi:10.1126/scitranslmed.aaw4744. 33177180 PMC8519505

[133] Peterson LB , Bell CJM, Howlett SK, Pekalski ML, Brady K, Hinton H, et al. A long-lived IL-2 mutein that selectively activates and expands regulatory T cells as a therapy for autoimmune disease. J Autoimmun 2018, 95, 1–14. doi:10.1016/j.jaut.2018.10.017. 30446251 PMC6284106

[134] Khoryati L , Pham MN, Sherve M, Kumari S, Cook K, Pearson J, et al. An IL-2 mutein engineered to promote expansion of regulatory T cells arrests ongoing autoimmunity in mice. Sci Immunol 2020, 5, eaba5264. doi:10.1126/sciimmunol.aba5264. 32817295 PMC7643170

[135] Dixit N , Fanton C, Langowski JL, Kirksey Y, Kirk P, Chang T, et al. NKTR-358: a novel regulatory T-cell stimulator that selectively stimulates expansion and suppressive function of regulatory T cells for the treatment of autoimmune and inflammatory diseases. J Transl Autoimmun 2021, 4, 100103. doi:10.1016/j.jtauto.2021.100103. 34041473 PMC8141531

[136] Spangler JB , Tomala J, Luca VC, Jude KM, Dong S, Ring AM, et al. Antibodies to interleukin-2 elicit selective T cell subset potentiation through distinct conformational mechanisms. Immunity 2015, 42, 815–25. doi:10.1016/j.immuni.2015.04.015. 25992858 PMC4439582

[137] Trotta E , Bessette PH, Silveria SL, Ely LK, Jude KM, Le DT, et al. A human anti-IL-2 antibody that potentiates regulatory T cells by a structure-based mechanism. Nat Med 2018, 24, 1005–14. doi:10.1038/s41591-018-0070-2. 29942088 PMC6398608

[138] Ward NC , Yu A, Moro A, Ban Y, Chen X, Hsiung S, et al. IL-2/CD25: a long-acting fusion protein that promotes immune tolerance by selectively targeting the IL-2 receptor on regulatory T cells. J Immunol 2018, 201, 2579–92. doi:10.4049/jimmunol.1800907. 30282751 PMC6200646

[139] Harris KE , Lorentsen KJ, Malik-Chaudhry HK, Loughlin K, Basappa HM, Hartstein S, et al. A bispecific antibody agonist of the IL-2 heterodimeric receptor preferentially promotes in vivo expansion of CD8 and NK cells. Sci Rep 2021, 11, 10592. doi:10.1038/s41598-021-90096-8. 34011961 PMC8134639

[140] Schreiber TH , Wolf D, Tsai MS, Chirinos J, Deyev VV, Gonzalez L, et al. Therapeutic Treg expansion in mice by TNFRSF25 prevents allergic lung inflammation. J Clin Invest 2010, 120, 3629–40. doi:10.1172/JCI42933. 20890040 PMC2947231

[141] Wolf D , Schreiber TH, Tryphonopoulos P, Li S, Tzakis AG, Ruiz P, et al. Tregs expanded in vivo by TNFRSF25 agonists promote cardiac allograft survival. Transplantation 2012, 94, 569–74. doi:10.1097/TP.0b013e318264d3ef. 22902792

[142] Marfil-Garza BA , Pawlick RL, Szeto J, Kroger C, Tahiliani V, Hefler J, et al. Tumor necrosis factor receptor superfamily member 25 (TNFRSF25) agonists in islet transplantation: Endogenous in vivo regulatory T cell expansion promotes prolonged allograft survival. Am J Transplant 2022, 22, 1101–14. doi: 10.1111/ajt.16940. 34965021

[143] Gorczynski RM , Sadozai H, Zhu F, Khatri I. Effect of infusion of monoclonal antibodies to tumour necrosis factor-receptor super family 25 on graft rejection in allo-immune mice receiving autologous marrow transplantation. Immunology 2017, 150, 418–31. doi:10.1111/imm.12693. 27859243 PMC5343351

[144] Mavers M , Simonetta F, Nishikii H, Ribado JV, Maas-Bauer K, Alvarez M, et al. Activation of the DR3-TL1A axis in donor mice leads to regulatory T cell expansion and activation with reduction in graft-versus-host disease. Front Immunol 2019, 10, 1624. doi:10.3389/fimmu.2019.01624. 31379829 PMC6652149

[145] Kim B-S , Nishikii H, Baker J, Pierini A, Schneidawind D, Pan Y, et al. Treatment with agonistic DR3 antibody results in expansion of donor Tregs and reduced graft-versus-host disease. Blood 2015, 126, 546–57. doi:10.1182/blood-2015-04-637587. 26063163 PMC4513255

[146] Wolf D , Bader CS, Barreras H, Copsel S, Pfeiffer BJ, Lightbourn CO, et al. Superior immune reconstitution using Treg-expanded donor cells versus PTCy treatment in preclinical HSCT models. JCI Insight 2018, 3(20), 1–14. doi:10.1172/jci.insight.121717. PMC6237457 30333311

[147] Torrey H , Kühtreiber WM, Okubo Y, Tran L, Case K, Zheng H, et al. A novel TNFR2 agonist antibody expands highly potent regulatory T cells. Sci Signaling 2020, 13, eaba9600. doi:10.1126/scisignal.aba9600. 33293464

[148] Padutsch T , Sendetski M, Huber C, Peters N, Pfizenmaier K, Bethea JR, et al. Superior Treg-expanding properties of a novel dual-acting cytokine fusion protein. Front Pharmacol 2019, 10, 1490. doi:10.3389/fphar.2019.01490. 31920671 PMC6930692

[149] Mayes PA , Hance KW, Hoos A. The promise and challenges of immune agonist antibody development in cancer. Nat Rev Drug Discovery 2018, 17, 509–27. doi:10.1038/nrd.2018.75. 29904196

[150] Jamison BL , Dilisio JE, Beard KS, Neef T, Bradley B, Goodman J, et al. Tolerogenic delivery of a hybrid insulin peptide markedly prolongs islet graft survival in the NOD mouse. Diabetes 2022, 71, 483–96. doi:10.2337/db20-1170. 35007324 PMC8893950

[151] Zhou H , Lou F, Bai J, Sun Y, Cai W, Sun L, et al. A peptide encoded by pri-miRNA-31 represses autoimmunity by promoting T_reg_ differentiation. EMBO Rep 2022, 23(5), 1–20. doi:10.15252/embr.202153475. PMC9066071 35343645

[152] Zhang L , Ke F, Liu Z, Bai J, Liu J, Yan S, et al. MicroRNA-31 negatively regulates peripherally derived regulatory T-cell generation by repressing retinoic acid-inducible protein 3. Nat Commun 2015, 6, 7639. doi:10.1038/ncomms8639. 26165721 PMC4510656

